# The mediating role of resilience on the association between family satisfaction and lower levels of depression and anxiety among Chinese adolescents

**DOI:** 10.1371/journal.pone.0283662

**Published:** 2023-05-25

**Authors:** Beizhu Ye, Joseph T. F. Lau, Ho Hin Lee, Jason C. H. Yeung, Phoenix K. H. Mo

**Affiliations:** 1 Department of Social Medicine and Health Management, School of Public Health, Zhengzhou University, Zhengzhou, China; 2 Center for Health Behaviours Research, JC School of Public Health and Primary Care, Faculty of Medicine, The Chinese University of Hong Kong, Hong Kong, Hong Kong; 3 School of Mental Health, Wenzhou Medical University, Wenzhou, China; 4 Zhejiang Provincial Clinical Research Center for Mental Disorders, The Affiliated Wenzhou Kangning Hospital, Wenzhou, China; University of Catania Libraries and Documentation Centre: Universita degli Studi di Catania, ITALY

## Abstract

**Purpose:**

This study aimed to explore the association between family satisfaction, resilience, and anxiety and depression among adolescents, and the mediating role of resilience in these relationships.

**Methods:**

A cross-sectional study was conducted among grade 8 to 9 students from 4 secondary schools in Hong Kong. A total of 1,146 participants completed the survey.

**Results:**

Respectively 45.8% and 58.0% of students scored above the cut-off for mild anxiety and mild depression. Results from linear regression analyses showed that family satisfaction was positively associated with resilience, and both family satisfaction and resilience were and negatively associated with anxiety and depression. The mediating effects of resilience on the relationship between family satisfaction and anxiety/ depression (26.3% and 31.1% effects accounted for, respectively) were significant.

**Conclusions:**

Both family satisfaction and resilience have important influence on adolescent mental health. Interventions that seek to promote positive family relationships and resilience of adolescents may be effective in preventing and reducing anxiety and depression symptoms among adolescents.

## Introduction

Mental health problems, with most of them having a relatively young onset, have significantly affected children and adolescents worldwide. Anxiety and depression are the most common mental health problems reported, and they are the top ten causes of disability-adjusted life-years for younger population aged 10–24 years [1–3]. A sharp increase in anxiety and depression has been observed over the past few decades [4, 5]. In Hong Kong, a representative study among 9,518 secondary school students showed that the level of depression was at moderate or severe level as measured by the Center for Epidemiological Studies-Depression (CES-D) [6]. Another study among 3,136 secondary school students in Hong Kong showed that respectively 54.3% and 65.8% of males and females scored above the cut-off for mild depression, as measured by the CES-D [7].

Anxiety and depression are associated with significant adverse consequences among adolescents, such as substance abuse, poor physical health, underachievement in schools, harmful social outcomes, subsequent depression in later life, and higher risk of suicide [8, 9]. Moreover, they may induce long-term effects throughout life, and even affect the mental health of offspring [10–13]. Accounting for the largest population in the world, China suffers from a huge burden of mental health problems. Understanding the factors underlying the occurrence of mental problems is greatly warranted.

Adolescents are at a critical period of physical and mental development. They constantly strive for independence but simultaneously may encounter many physical and psychosocial challenges as they cope with the tremendous stressors in school and social lives. Studies have shown that adolescents’ anxiety and depression are subject to the influence of various individual and social factors [14–18]. Among them, the influence of family on adolescent mental health is instrumental [19–21]. Specifically, family satisfaction, which measures the extent to which one is satisfied with one’s family’s overall functioning and relationships, is one of the most influential aspects of life satisfaction. Studies have shown that higher family satisfaction leads to lower levels of worry, anxiety, and depression in adolescents [22]. Both cross-sectional and longitudinal studies have proven that family satisfaction was a significant protective factor of anxiety and depression among adolescents [23–25]. One study in South America found that family satisfaction and communication explained 18% of the variance in child anxiety [26].

Despite the strong link between family satisfaction and mental health problems established in the literature, very little is known about how the association between family satisfaction and mental health problems can be conceptualized. The present study proposed that resilience may be an important mediator that help to clarify the association between family satisfaction and anxiety/ depression. Psychological resilience commonly pertains to the capacity of an individual to survive and adapt to difficult situations [27, 28]. Individuals who are resilient possess the ability to effectively cope with adversities and stress while maintaining normal mental and physical functioning [29, 30]. A dynamic systems perspective of resilience has been reflected recently, conceptualizing it as the capacity of dynamic systems to adapt to adversities and threats [31]. Resilience was found to be strongly associated with mental health in children and adolescents, contributing to positive outcomes, and serving as protective factor within individuals’ well-being [32, 33]. The pivotal role of resilience in saving individuals from mental disorders amid violent and life-threatening events was also demonstrated in the literature [34, 35]. Furthermore, school-based resilience-focused interventions were effective in reducing depressive symptoms, internalizing problems, externalizing problems, and general psychological distress among children and adolescents [36, 37].

Family is also a fundamental source of support and an influential factor of resilience for adolescents. Adolescents who have a positive family environment might be more likely to acquire the skills in coping with stressors and establishing a positive personal development. Studies have reported that positive family factors, such as lower levels of parental psychological control and maternal overprotection, high levels of maternal warmth, greater family cohesion, and easier temperament were associated with greater odds of resilience in youths [38, 39]. Masten’s multisystem resilience approach also included a multitude of crucial family attributes, such as sensitive caregiving, family cohesion, sense of belonging, and family management in facilitating adolescent resilience [31]. Nevertheless, very few studies have examined the role of family satisfaction on resilience in the Chinese context.

The consistent empirical evidence established between resilience, family satisfaction and mental health suggested that resilience could be a potential mediator of the association between life satisfaction and mental health. Meanwhile, extant theories also lent support to the potential mediating role of resilience. According to the Resiliency Theory, two types of promotive factors operate to ameliorate risk factors and mental ill health: positive factors within individuals and resources available outside the individuals [40, 41]. In this regard, family satisfaction could serve as an important positive resource that promotes resilience, which further buffer the negative effects of stress of mental illness. Growing evidence on the mediating role of resilience on mental health has been reported in literature [42–44]. For example, a study among 2,925 medical students in China found that resilience mediated the relationship between personality traits and anxiety [45]. Another recent study among 1,845 adolescents in China also found that resilience mediated the link of subjective family socioeconomic status with life satisfaction [46]. However, to the best of our knowledge, the possible mediating role of resilience in the association between family satisfaction on anxiety and depression has not yet been investigated.

Considering the significant mental health problems documented among Chinese adolescents, there is an urgent need to understand the factors associated with their mental health so that appropriate interventions could be designed. With respect to the indispensable role of family factors and resilience on adolescent mental health, it is expected that findings of the present study would provide important directions and implications on the design of mental health interventions for Chinese adolescents. The present study aimed to explore the association between family satisfaction and resilience on mental health problems (i.e. anxiety and depression) among adolescents in Hong Kong. It also examined whether resilience mediated the relationship between family satisfaction and anxiety/ depression. We hypothesized that family satisfaction would be associated with higher levels of resilience, which in turn, would be associated with lower levels of anxiety/ depression among adolescents.

## Methods

### Study design

A cross-sectional study was conducted among secondary school students in Hong Kong. Invitation letters were sent to all secondary schools of Hong Kong, four replied and showed interest in taking part in the study. Discussions were made with the school management team, it was agreed that secondary two and three students (i.e. grade eight and nine) would be invited as secondary one students have just transited to secondary school and secondary four to six students would need to prepare for public examinations. An opt-out parental informed consent was used, in which parents were asked to return a signed form if they refused to let their students take part in the survey. No parents returned such forms. In a classroom setting and with the absence of teachers, research assistants briefed the students the objectives of the study and the logistics that would be involved. Participants were informed that their participation was voluntary and return of the questionnaire implied informed consent. They were also guaranteed that the data would be kept confidential and only be accessible by the researchers of the team. Students then self-administered the questionnaire which took about 20 minutes to complete. The questionnaire was sent to a total of 1,185 students, and all of them returned the questionnaire. Ethics approval was obtained from the Survey and Behavioral Ethics Committee of the institution of the Chinese University of Hong Kong.

As a measure of quality control, 39 cases with at least one scale having more than 20% of its items missing were excluded from data analysis. The final sample consisted of 1,146 participants.

### Measures

*Anxiety* was assessed by the 7-item General Anxiety Disorder (GAD) Scale [47]. It has been validated among Chinese adolescents [48]. Each item was rated on a 4-point scale (0 = not at all to 3 = almost all of the time). The total score ranges from 0 to 21, and higher scores indicate more severe anxiety symptoms. The Cronbach’s alpha was 0.941 in the present study. The GAD scores of ≥5, 10, and 15 are defined as mild, moderate, and severe anxiety respectively.

*Depression* was measured by the Chinese version of 20-item CES-D [49]. It has been validated among Chinese adolescents [50, 51]. Participants were inquired about how often they experienced the feelings in the past week. Each item is rated on a 4-point scale (0 = rarely or none of the time to 3 = almost or all of the time). The total score ranges from 0 to 60, and higher scores indicate more severe depressive symptoms. The Cronbach’s alpha was 0.905 in the present study. The CES-D scores of ≥16, 21 and 25 are defined as mild, moderate, and severe depression respectively.

*Family satisfaction* was measured by the 10-item Family Satisfaction Scale, which measures one’s level of satisfaction with family overall functioning and relationships [52]. It has been used among adolescents [53, 54]. Each item is rated on a 5-point scale (1 = very dissatisfied to 5 = very satisfied). The total score ranges from 10 to 50, and higher scores indicate greater satisfaction with the family. The Cronbach’s alpha was 0.962 in the present study.

*Resilience* was assessed by the 25-item Connor-Davidson Resilience Scale [55]. It has been validated among Chinese adolescents [56]. Participants were asked how often they experienced various situations on a 5-point scale (1 = not at all to 5 = almost all of the time). The total score ranges from 25 to 125, and higher scores indicate higher level of resilience. The Cronbach’s alpha was 0.939 in the present study.

*Socio-demographic variables* included age (as a continuous variable), sex (male / female), grade (eighth / ninth grade), and housing type (public housing / Home Ownership Scheme (HOS) / private housing / others).

### Statistical analysis

The missing values from all of the items ranged from 0.1% to 0.4%, which were imputed by using the modal value of the other items of the same scale. Descriptive statistics were presented. Continuous variables are presented as means ± standard deviations, and categorical variables are shown as percentages. Correlation between the variables under studied was calculated. To test the association between resilience and family satisfaction on anxiety/ depression, a series of hierarchical linear regression models were performed. Model 1 included socio-demographic variables only; model 2 added family satisfaction; and model 3 added resilience. Standardized regression coefficient (*β*), F, R^2^ and R^2^-changes (ΔR^2^) for each regression model were provided.

In addition, asymptotic and resampling strategies developed by Preacher and Hayes [57] were employed to test the significance of the mediating role of resilience on the associations between family satisfaction and anxiety/ depression. In the regression equations, family satisfaction was modeled as predictors, anxiety/ depression as the dependent variable, resilience as the mediator, and socio-demographic variables as covariates. The path coefficients a (family satisfaction to resilience), b (direct effects of resilience on anxiety/ depression), a*b products (indirect effects of family satisfaction on anxiety/ depression through resilience), c (total effects of family satisfaction on anxiety/ depression) and c’ (direct effects of family satisfaction on anxiety/ depression) were presented. We applied bootstrapping methods to obtain a 95% confidence interval (CI) for the mediated effects. The bootstrap estimate was based on 5,000 bootstrap samples, and the bias-corrected and accelerated 95% confidence interval (BCa95%CI) of indirect effect (a*b product) excluding 0 indicated a significant mediating role. We conducted all analyses using SPSS 22.0 (SPSS Inc., Chicago, IL). Statistical tests were two-tailed, and differences were considered significant when *p* <0.05.

## Results

### Socio-demographic characteristics and mental health status

The socio-demographic characteristics and mental health status of the participants are shown in Table 1. Average age was 13.58 years old (SD = 0.88), ranging from 11 to 18. Among the 1,146 students, 644 (56.89%) were males.

**Table 1:** Background characteristics of participants (N = 1,146).

Variable	N (%)	M (SD)
Age in years (Mean, SD)	13.58 (0.88)	
Sex		
Male	644 (56.89)	
Female	488 (43.11)	
Grade		
Eighth grade	546 (47.64)	
Ninth grade	600 (52.36)	
Housing type		
Public housing	434 (38.27)	
Home Ownership Scheme (HOS)	198 (17.46)	
Private housing	478 (42.15)	
Others	24 (2.12)	
Anxiety score		5.04 (5.30)
Normal	621(54.19)	
Mild	324(28.27)	
Moderate	126(10.99)	
Severe	75(6.54)	
Depression score		19.37 (11.08)
Normal	481(41.97)	
Mild	178(15.53)	
Moderate	143(12.48)	
Severe	344(30.02)	
Resilience score		82.10 (15.77)
Family satisfaction score		33.26 (9.27)

N: number, M(SD): Mean (standard deviation)

The average score of depression and anxiety of the current sample revealed a concerning mental health issue. Respectively 45.8% (49.2% for female and 42.9% for male, *p* = 0.022) and 58.0% of students (61.9% for female and 55.1% for male, *p* = 0.034) scored above the cut-off for mild anxiety and mild depression.

### Correlations among major variables

Results of Pearson correlation analysis are shown in Table 2. Family satisfaction was positively correlated with resilience (r = 0.42, *p*<0.001) and negatively correlated with anxiety (r = -0.29, *p*<0.001) and depression (r = -0.38, *p*<0.001). Resilience was negatively correlated with anxiety (r = -0.28, *p*<0.001) and depression (r = -0.41, *p*<0.001). The results were consistent to our hypothesized relationship among the variables.

**Table 2:** Correlations among major variables.

Variables	1	2	3	4
1. Family satisfaction	-			
2. Resilience	0.42***	-		
3. Anxiety	-0.29***	-0.28***	-	
4. Depression	-0.39***	-0.41***	0.72***	-

***p < 0.001 (2-tailed)

### Linear regression model for anxiety

Results of the hierarchical linear regression models of anxiety are presented in Table 3. Among the socio-demographic characteristics, female gender was positively related to anxiety (*β* = 0.07, *p*<0.05). Also, living in private houses was negatively associated with depression (*β* = -0.08, *p*<0.05). After controlling for these socio-demographic characteristics, family satisfaction (*β* = -0.28, *p*<0.001) was negatively associated with anxiety in Model 2. It remained significant (*β* = -0.20, *p*<0.001) after resilience (*β* = -0.18, *p*<0.001) was added in Model 3. Overall the model explains 12% of the variance in anxiety.

**Table 3:** Hierarchical linear regression analyses for anxiety and depression.

	Anxiety			Depression		
Variable	Model 1 (*β*)	Model 2 (*β*)	Model 3 (*β*)	Model 1 (*β*)	Model 2 (*β*)	Model 3 (*β*)
Age	0.05	0.03	0.03	0.07	0.05	0.05
Sex	0.07*	0.07*	0.05	0.12***	0.12***	0.09***
Grade	0.01	0.00	-0.01	-0.03	-0.04	-0.05
Housing type						
Home Ownership Scheme vs Public housing	-0.03	-0.02	0.00	-0.09**	-0.07*	-0.04
Private housing vs Public housing	-0.08*	-0.05	-0.03	-0.11**	-0.07*	-0.04
Others vs Public housing	-0.06	-0.04	-0.03	-0.06*	-0.04	-0.03
Family satisfaction		-0.28***	-0.20***		-0.37***	-0.25***
Resilience			-0.18***			-0.26***
F	2.97	15.92	18.47	5.96	32.05	42.62
R^2^	0.02	0.09	0.12	0.03	0.17	0.23
Change in R^2^	0.02	0.07	0.03	0.03	0.14	0.07

*p < 0.05, **p < 0.01, ***p < 0.001

### Linear regression model for depression

Results of the hierarchical linear regression models of depression are presented in Table 3. Similarly, among the socio-demographic characteristics, female gender was positively related to anxiety (*β* = 0.12, *p*<0.001). Also, compared with students living in public housing, those living in The Home Ownership Scheme (HOS) (*β* = -0.09, *p*<0.01), private houses (*β* = -0.11, *p*<0.01), and others (*β* = -0.06, *p*<0.05), were negatively associated with depression. After controlling for socio-demographic characteristics, family satisfaction was negatively associated with depression (*β* = -0.37, *p*<0.001) in Model 2. It remained significant (*β* = -0.25, *p*<0.001) after resilience (*β* = -0.26, *p*<0.001) was added in Model 3. Overall the model explains 23% of the variance in depression.

### The mediating role of resilience on anxiety/ depression

Table 4 shows the path coefficients a (family satisfaction to resilience) and b (direct effects of resilience on anxiety/ depression), a*b products (indirect effects of family satisfaction on anxiety/ depression through resilience) and BCa95%CI, coefficients c (total effects of family satisfaction on anxiety/ depression) and c’ (direct effects of family satisfaction on anxiety/ depression). For the model, family satisfaction (a = 0.70, *p*<0.001) was positively associated with resilience, and resilience (b = -0.06, *p*<0.001; b = -0.20, *p*<0.001, respectively) was negatively associated with anxiety and depression. The mediating effects of resilience on the relationship between family satisfaction and anxiety and depression were significant (a*b = -0.04, BCa95%CI: -0.06, -0.03; a*b = -0.14, BCa95%CI: -0.18, -0.11, respectively). The proportions of mediating roles (indirect effect/ total effect) of resilience on anxiety and depression were 26.3% and 31.1%, respectively.

**Table 4:** Mediating role of resilience on the association between family satisfaction and anxiety/ depression.

	Path coefficients				a*b (*BCa 95%CI*)
	C	A	b	c′	
Family satisfaction to anxiety	-0.16***	0.70***	-0.06***	-0.12***	-0.04*** (-0.06, -0.03)
Family satisfaction to depression	-0.45***	0.70***	-0.20***	-0.30***	-0.14*** (-0.18, -0.11)

*: *p* < 0.05, **: *p* < 0.01, ***: *p* < 0.001

c: total effects of family satisfaction on anxiety/ depression

a: effect of family satisfaction on resilience

b: direct effects of resilience on anxiety/ depression

c′: direct effects of family satisfaction on anxiety/ depression

a*b: indirect effects of family satisfaction on anxiety/ depression through resilience

BCa95%CI: bias-corrected and accelerated 95% confidence interval.

## Discussion

The present study explored the associations between family satisfaction, resilience, and anxiety/ depression among secondary school students in Hong Kong. First of all, it is important to note that 45.8% of our sample scored higher that the cut-off for mild anxiety, which was higher than 22.7% reported among adolescents in the USA [58]. Also, 58.0% scored higher than cut-off for mild depression, which was similar to that reported in a large representative study among secondary school students in Hong Kong (about 60%) [6], but higher than the counterparts in other countries such as mainland China (34.4%) [59] and Australia (38.3%) [60]. Findings were consistent with a previous cross-cultural study that reported a higher level of psychological distress among individuals in Hong Kong than other countries [61]. As Chinese culture emphasizes self-control, obedience and respect for authority, Chinese adolescents may be more vulnerable to developing internalizing pathology [62, 63]. A strong emphasis on academic performance, high levels of parental expectation and control, heavy school workload and intensive extra-curricular activities might also lead to high levels of stress among Hong Kong adolescents [64]. Furthermore, females were more vulnerable to anxiety and depression in our sample, which is consistent with the findings reported in the general population [65–67]. Overall, findings of the present study highlight an urgent need for interventions to reduce the prevalent mental health problems of adolescents in Hong Kong.

Greater family function and relationships are associated lower scores of anxiety and depression among adolescents in the literature. Our results showed that family satisfaction was a protective factor to adolescents’ anxiety and depression, which is in line with prior findings [23, 26]. In the present study, the family satisfaction score of our participants was comparable to the study in South America assessed by the same scale (M = 34.2, SD = 6.6) [26]. A review conducted by Bögels & Brechman-Toussaint revealed that poor family functioning such as marital conflict, and co-parenting and parental rearing strategies, predicted child anxiety [68]. Moreover, other studies suggested that integrating family into intervention and treatment of depression among adolescents received greater improvement in functioning, hopelessness and self-reported depression than traditional treatment [69, 70]. Given the critical importance of families in adolescents’ mental health, empowering families to enact adaptive changes and involvement in intervention should be considered.

Apart from family satisfaction, our findings also reported that resilience was another protective factor of adolescents’ anxiety and depression, which is consistent to the extant literature [31, 33, 71, 72]. Resilience can be viewed as a defense mechanism, which enables people to thrive in the face of adversities [73]. Therefore, resilience may be an essential asset that should be promoted for prevention and treatment of mental health problems.

We found that resilience was associated with family satisfaction, which has not been previously tested in the Chinese context. Such findings corroborate with existing studies that family communication, cohesion and close relationships were positively associated with resilient outcomes in youth among the western population [38, 39, 74]. Furthermore, our results highlighted a significant indirect pathway by which resilience mediated the relationship between family satisfaction and anxiety/ depression. In other words, family satisfaction was associated with higher levels of resilience, which in turn, linked with lower levels of anxiety and depression among adolescents. Based on the bootstrap method, the proportion of mediating roles of resilience on anxiety and depression was 26.3% and 31.1%, respectively. Considering the complexity of mediators between family satisfaction and mental health problems, it is not surprising to find resilience as a partial but not full mediation. To our knowledge, this is the first study to test the potential mediating role of resilience in the effect of family satisfaction on anxiety and depression. Findings offer theoretical support to the dynamic systems perspective of resilience and the Resiliency Theory which posit that social factors, such as family support and family satisfaction, can be useful promotive factors that promote resilience and reduce anxiety/ depression. Adolescents who are satisfied with their family environment might be may be more capable of developing positive coping strategies that can act against the potential harm of stressors and help them manage adversity more effectively. These can further protect them from the detrimental effects of mental illness. The results are also consistent with a study among individuals with multiple sclerosis, which showed that resilience mediated the relationships between social support from family members and subsequent mental health outcomes such as depressive and anxious symptomatology [75]. The importance of engaging family in cultivating resilience and mental health of adolescents is underscored.

### Implications

Findings of the present study would provide important implications for practice. Identifying the mediator of the association between family satisfaction and resilience would be particularly useful as it provides insights on the complex association between the variables, which allows health care professionals to understand the focal point and tailor appropriate strategies for effective mental health promotion. In view of the significant mediating effect of resilience, resilience programs may be a useful intervention in easing adolescents’ anxiety and depression symptoms. A randomized control trial conducted in the USA indicated that the resiliency intervention group had significantly higher scores in effective coping strategies, lower scores on depressive symptoms, and less perceived stress than the control group [76]. Similar resiliency interventions also resulted in lower levels of mental health symptoms among populations in Australia and Thailand [77, 78]. These programs promote development of personal psychological attributes such as optimism, self-efficacy and adaptability, and equip adolescents with positive coping methods as a means to promote their resilience and mental health. They also. The mediating role of resilience also suggest that monitoring adolescents’ resilience may be a good indicator for health care professors to identify emerging adolescents who are at risk for mental ill health. Resilience should be considered as a viable means to track the changes in mental health of adolescents across time.

Additionally, identification of specific factors that promote resilience may also be effective in promoting mental health among adolescents. The significant association between family satisfaction and anxiety/ depression highlights the importance of involving family members in mental health promotion among adolescents. Interventions that promote family management skills, positive family interaction, and effective family communications would be effective in reducing stress and family conflict and consequently, anxiety and depression of adolescents [79, 80]. Supporting families to create a safe and supportive environment where they can openly discuss their feelings and worries, and providing information and resources about mental health to parents can also help them better understand and support their adolescents in cultivating positive mental health.

### Limitations

There are some limitations in the present study that should be mentioned. First, although we delineated the potential mechanism which resilience and family satisfaction were associated with anxiety and depression, the cross-sectional design precluded the opportunity to draw causal conclusions. Prospective research is warranted to confirm our findings. Second, anxiety and depression were assessed through self-reported questionnaires, rather than clinical diagnosis. However, both the GAD and CES-D are validated and commonly used to screen probable anxiety and depression among adolescents [81, 82]. Third, data was collected from grade 8 and 9 students from 4 secondary schools, findings might not be generalizable to other secondary school students in Hong Kong. Finally, it is acknowledged that other aspects of satisfaction, such as social and life satisfaction, would also have an important influence on adolescent resilience and mental health [83, 84]. The present study only measured the role of family satisfaction on mental health among adolescents. It is a limitation that other aspects of satisfaction have not been captured in the present study.

## Conclusions

In summary, we identified a very high prevalence of probable anxiety and depression among secondary school students in Hong Kong. Family satisfaction has a direct negative association with anxiety and depression and may also strengthen resilience, which in turn, reduce anxiety and depression symptoms. Both family satisfaction and resilience have instrumental influence on adolescent mental health, therefore, promoting positive family relationships and resilience of adolescents may be an effective strategy in preventing and alleviating anxiety and depression symptoms among adolescents.

## References

[1] Collaborators GDaI. Global burden of 369 diseases and injuries in 204 countries and territories, 1990–2019: a systematic analysis for the Global Burden of Disease Study 2019. Lancet. 2020;396(10258):1204–22. doi: 10.1016/S0140-6736(20)30925-9 33069326 PMC7567026

[2] Fusar-Poli P. Integrated Mental Health Services for the Developmental Period (0 to 25 Years): A Critical Review of the Evidence. Front Psychiatry. 2019;10:355. doi: 10.3389/fpsyt.2019.00355 31231250 PMC6567858

[3] Kessler RC, Berglund P, Demler O, Jin R, Merikangas KR, Walters EE. Lifetime prevalence and age-of-onset distributions of DSM-IV disorders in the national comorbidity survey replication.(vol 62, pg 593, 2005). Arch Gen Psychiat. 2005;62(7):768–. 10.1001/archpsyc.62.6.593 15939837

[4] Beesdo K, Knappe S, Pine DS. Anxiety and Anxiety Disorders in Children and Adolescents: Developmental Issues and Implications for DSM-V. Psychiat Clin N Am. 2009;32(3):483–524. doi: 10.1016/j.psc.2009.06.002 19716988 PMC3018839

[5] Thapar A, Collishaw S., Pine D. S., & Thapar A. K. Depression in adolescence. The Lancet. 2012;379 (9820):1056–67. doi: 10.1016/S0140-6736(11)60871-4 22305766 PMC3488279

[6] Wu AMS, Li JB, Lau JTF, Mo PKH, Lau MMC. Potential impact of internet addiction and protective psychosocial factors onto depression among Hong Kong Chinese adolescents—direct, mediation and moderation effects. Compr Psychiat. 2016;70:41–52. doi: 10.1016/j.comppsych.2016.06.011 27624422

[7] She R, Wong K, Lin J, Leung K, Zhang Y, Yang X. How COVID-19 stress related to schooling and online learning affects adolescent depression and Internet gaming disorder: Testing Conservation of Resources theory with sex difference. J Behav Addict. 2021;10(4):953–66. doi: 10.1556/2006.2021.00069 34665762 PMC8987435

[8] Ranasinghe S, Ramesh S, Jacobsen KH. Hygiene and mental health among middle school students in India and 11 other countries. J Infect Public Heal. 2016;9(4):429–35. doi: 10.1016/j.jiph.2015.11.007 26655444

[9] Johnson D, Dupuis G, Piche J, Clayborne Z, Colman I. Adult mental health outcomes of adolescent depression: A systematic review. Depression and Anxiety. 2018;35(8):700–16. doi: 10.1002/da.22777 29878410

[10] Avenevoli S, Merikangas KR. Implications of high-risk family studies for prevention of depression. Am J Prev Med. 2006;31(6):S126–S35. doi: 10.1016/j.amepre.2006.07.003 17175407

[11] Collishaw S, Hammerton G, Mahedy L, Sellers R, Owen MJ, Craddock N, et al. Mental health resilience in the adolescent offspring of parents with depression: a prospective longitudinal study. The lancet Psychiatry. 2016;3(1):49–57. doi: 10.1016/S2215-0366(15)00358-2 26654748 PMC4703896

[12] Vismara L, Sechi C, Lucarelli L. Fathers’ and mothers’ depressive symptoms: internalizing/externalizing problems and dissociative experiences in their adolescent offspring. Current Psychology. 2022;41(1):247–57.

[13] Clayborne ZM, Varin M, Colman I. Systematic Review and Meta-Analysis: Adolescent Depression and Long-Term Psychosocial Outcomes. Journal of the American Academy of Child & Adolescent Psychiatry. 2019;58(1):72–9. doi: 10.1016/j.jaac.2018.07.896 30577941

[14] Cheng Y, Li XC, Lou CH, Sonenstein FL, Kalamar A, Jejeebhoy S, et al. The Association Between Social Support and Mental Health Among Vulnerable Adolescents in Five Cities: Findings From the Study of the Well-Being of Adolescents in Vulnerable Environments. J Adolescent Health. 2014;55(6):S31–S8. 10.1016/j.jadohealth.2014.08.020 25454000

[15] Lamb DJ, Middeldorp CM, van Beijsterveldt CEM, Bartels M, van der Aa N, Polderman TJC, et al. Heritability of Anxious-Depressive and Withdrawn Behavior: Age-Related Changes During Adolescence. J Am Acad Child Psy. 2010;49(3):248–55. 20410714

[16] Mathews BL, Koehn AJ, Abtahi MM, Kerns KA. Emotional Competence and Anxiety in Childhood and Adolescence: A Meta-Analytic Review. Clin Child Fam Psych. 2016;19(2):162–84. doi: 10.1007/s10567-016-0204-3 27072682

[17] Niarchou M, Zammit S, Lewis G. The Avon Longitudinal Study of Parents and Children (ALSPAC) birth cohort as a resource for studying psychopathology in childhood and adolescence: a summary of findings for depression and psychosis. Soc Psych Psych Epid. 2015;50(7):1017–27. doi: 10.1007/s00127-015-1072-8 26002411

[18] Rueger SY, Malecki CK, Pyun Y, Aycock C, Coyle S. A Meta-Analytic Review of the Association Between Perceived Social Support and Depression in Childhood and Adolescence. Psychol Bull. 2016;142(10):1017–67. doi: 10.1037/bul0000058 27504934

[19] Carr A. The handbook of child and adolescent clinical psychology: a contextual approach. London: Routledge; 2006.

[20] Kienhorst I, Harrington R. Depressive disorder in childhood and adolescence. Kind en adolescent. 2000;21(1):38–9.

[21] Rohner RP, Britner PA. Worldwide mental health correlates of parental acceptance-rejection: Review of cross-cultural and intracultural evidence. Cross-Cult Res. 2002;36(1):16–47.

[22] Proctor C, Linley PA. Life Satisfaction in Youth. Increasing Psychological Well-Being in Clinical and Educational Settings: Interventions and Cultural Contexts. 2014;8:199–215.

[23] Vulic-Prtoric A, Macuka I. Family and coping factors in the differentiation of childhood anxiety and depression. Psychol Psychother-T. 2006;79:199–214. doi: 10.1348/147608305X52676 16774718

[24] Way N, Robinson MG. A longitudinal study of the effects of family, friends, and school experiences on the psychological adjustment of ethnic minority, low-SES adolescents. Journal of Adolescent Research. 2003;18:324–46.

[25] Magson NR, Freeman JYA, Rapee RM, Richardson CE, Oar EL, Fardouly J. Risk and Protective Factors for Prospective Changes in Adolescent Mental Health during the COVID-19 Pandemic. J Youth Adolesc. 2021;50(1):44–57. doi: 10.1007/s10964-020-01332-9 33108542 PMC7590912

[26] Nicolais CJ, Perrin PB, Panyavin I, Nicholls EG, Plaza SLO, Quintero LM, et al. Family dynamics and psychosocial functioning in children with SCI/D from Colombia, South America. J Spinal Cord Med. 2016;39(1):58–66. doi: 10.1179/2045772314Y.0000000291 25582185 PMC4725793

[27] Hart PL, Brannan JD, De Chesnay M. Resilience in nurses: an integrative review. Journal of nursing management. 2014;22(6):720–34. doi: 10.1111/j.1365-2834.2012.01485.x 25208943

[28] Luthar SS, Cicchetti D, Becker B. The construct of resilience: a critical evaluation and guidelines for future work. Child development. 2000;71(3):543–62. doi: 10.1111/1467-8624.00164 10953923 PMC1885202

[29] Wu G, Feder A, Cohen H, Kim JJ, Calderon S, Charney DS, et al. Understanding resilience. Frontiers in behavioral neuroscience. 2013;7:10. doi: 10.3389/fnbeh.2013.00010 23422934 PMC3573269

[30] Rutter M. Resilience as a dynamic concept. Development and psychopathology. 2012;24(2):335–44. doi: 10.1017/S0954579412000028 22559117

[31] Masten AS, Lucke CM, Nelson KM, Stallworthy IC. Resilience in Development and Psychopathology: Multisystem Perspectives. Annu Rev Clin Psychol. 2021;17(1):521–49. doi: 10.1146/annurev-clinpsy-081219-120307 33534615

[32] Ungar M, Theron L. Resilience and mental health: how multisystemic processes contribute to positive outcomes. The lancet Psychiatry. 2020;7(5):441–8. doi: 10.1016/S2215-0366(19)30434-1 31806473

[33] Mesman E, Vreeker A, Hillegers M. Resilience and mental health in children and adolescents: an update of the recent literature and future directions. Curr Opin Psychiatry. 2021;34(6):586–92. doi: 10.1097/YCO.0000000000000741 34433193 PMC8500371

[34] Horn SR, Charney DS, Feder A. Understanding resilience: New approaches for preventing and treating PTSD. Exp Neurol. 2016;284(Pt B):119–32. doi: 10.1016/j.expneurol.2016.07.002 27417856

[35] Thompson NJ, Fiorillo D, Rothbaum BO, Ressler KJ, Michopoulos V. Coping strategies as mediators in relation to resilience and posttraumatic stress disorder. J Affect Disord. 2018;225:153–9. doi: 10.1016/j.jad.2017.08.049 28837948 PMC5626644

[36] Dray J, Bowman J, Campbell E, Freund M, Wolfenden L, Hodder RK, et al. Systematic Review of Universal Resilience-Focused Interventions Targeting Child and Adolescent Mental Health in the School Setting. Journal of the American Academy of Child & Adolescent Psychiatry. 2017;56(10):813–24. doi: 10.1016/j.jaac.2017.07.780 28942803

[37] Leppin AL, Bora PR, Tilburt JC, Gionfriddo MR, Zeballos-Palacios C, Dulohery MM, et al. The Efficacy of Resiliency Training Programs: A Systematic Review and Meta-Analysis of Randomized Trials. Plos One. 2014;9(10). doi: 10.1371/journal.pone.0111420 25347713 PMC4210242

[38] Lewandowski RE, Verdeli H, Wickramaratne P, Warner V, Mancini A, Weissman M. Predictors of Positive Outcomes in Offspring of Depressed Parents and Non-depressed Parents Across 20 Years. J Child Fam Stud. 2014;23(5):800–11. doi: 10.1007/s10826-013-9732-3 25374449 PMC4217704

[39] Brennan PA, Le Brocque R, Hammen C. Maternal depression, parent-child relationships, and resilient outcomes in adolescence. J Am Acad Child Psy. 2003;42(12):1469–77. doi: 10.1097/00004583-200312000-00014 14627882

[40] Fergus S, Zimmerman MA. Adolescent resilience: a framework for understanding healthy development in the face of risk. Annu Rev Public Health. 2005;26:399–419. doi: 10.1146/annurev.publhealth.26.021304.144357 15760295

[41] Zimmerman MA, Stoddard SA, Eisman AB, Caldwell CH, Aiyer SM, Miller A. Adolescent Resilience: Promotive Factors That Inform Prevention. Child Development Perspectives. 2013;7(4):215–20. doi: 10.1111/cdep.12042 24288578 PMC3839856

[42] Shi M, Wang XX, Bian YG, Wang L. The mediating role of resilience in the relationship between stress and life satisfaction among Chinese medical students: a cross-sectional study. Bmc Med Educ. 2015;15. 10.1186/s12909-015-0297-2 PMC4332721 25890167

[43] Zhang LY, Li XM, Qiao S, Zhou YJ, Shen ZY, Tang ZZ, et al. The mediating role of individual resilience resources in stigma-health relationship among people living with HIV in Guangxi, China. Aids Care. 2015;27(10):1317–25. doi: 10.1080/09540121.2015.1054338 26274908 PMC6234005

[44] Tian X, Lu J, Che Y, Fang D, Ran H, He X, et al. Childhood maltreatment and self-harm in Chinese adolescents: moderation and mediation via resilience. BMC Public Health. 2021;21(1):1561. doi: 10.1186/s12889-021-11605-y 34404376 PMC8371889

[45] Shi M, Liu L, Wang ZY, Wang L. The Mediating Role of Resilience in the Relationship between Big Five Personality and Anxiety among Chinese Medical Students: A Cross-Sectional Study. Plos One. 2015;10(3). 10.1371/journal.pone.0119916 PMC4368674 25794003

[46] Yan W, Zhang L, Li W, Kong F. How is Subjective Family Socioeconomic Status Related to Life Satisfaction in Chinese Adolescents? The Mediating Role of Resilience, Self-Esteem and Hope. Child Ind Res. 2022;15(5):1565–81.

[47] Spitzer RL, Kroenke K, Williams JBW, Lowe B. A brief measure for assessing generalized anxiety disorder—The GAD-7. Arch Intern Med. 2006;166(10):1092–7. doi: 10.1001/archinte.166.10.1092 16717171

[48] Sun J, Liang K, Chi X, Chen S. Psychometric Properties of the Generalized Anxiety Disorder Scale-7 Item (GAD-7) in a Large Sample of Chinese Adolescents. Healthcare [Internet]. 2021; 9(12). 10.3390/healthcare9121709 PMC8701121 34946435

[49] Radloff LS. The CES-D Scale: A Self-Report Depression Scale for Research in the General Population. Applied Psychological Measurement. 1977;1(3):385–401.

[50] Chen Z-y, Yang X-d, Li X-y. Psychometric features of CES-D in Chinese adolescents. Chinese Journal of Clinical Psychology. 2009;17:443–5.

[51] Yang W, Xiong G, Garrido LE, Zhang JX, Wang MC, Wang C. Factor structure and criterion validity across the full scale and ten short forms of the CES-D among Chinese adolescents. Psychol Assess. 2018;30(9):1186–98. doi: 10.1037/pas0000559 29658726

[52] Olson D. Families, what makes them work?. Journal of Marriage & Family. 1983;46(2):501.

[53] Stavropoulos V, Lazaratou H, Marini E, Dikeos D. Low Family Satisfaction and Depression in Adolescence: The Role of Self-Esteem. Journal of Educational and Developmental Psychology. 2015;5:109–18.

[54] Scabini E, Galimberti C. Adolescents and young adults: a transition in the family. Journal of Adolescence. 1995;18(5):593–606.

[55] Connor KM, Davidson JRT. Development of a new resilience scale: The Connor-Davidson Resilience scale (CD-RISC). Depress Anxiety. 2003;18(2):76–82. doi: 10.1002/da.10113 12964174

[56] Yu X-n, Lau JTF, Mak WWS, Zhang J, Lui WWS, Zhang J. Factor structure and psychometric properties of the Connor-Davidson Resilience Scale among Chinese adolescents. Comprehensive Psychiatry. 2011;52(2):218–24. doi: 10.1016/j.comppsych.2010.05.010 21295229

[57] Preacher KJ, Hayes AF. Asymptotic and resampling strategies for assessing and comparing indirect effects in multiple mediator models. Behav Res Methods. 2008;40(3):879–91. doi: 10.3758/brm.40.3.879 18697684

[58] Copeland WE, Angold A, Shanahan L, Costello EJ. Longitudinal Patterns of Anxiety From Childhood to Adulthood: The Great Smoky Mountains Study. J Am Acad Child Psy. 2014;53(1):21–33. doi: 10.1016/j.jaac.2013.09.017 24342383 PMC3939681

[59] Ma L, Mazidi M, Li K, Li Y, Chen S, Kirwan R, et al. Prevalence of mental health problems among children and adolescents during the COVID-19 pandemic: A systematic review and meta-analysis. J Affect Disord. 2021;293:78–89. doi: 10.1016/j.jad.2021.06.021 34174475 PMC9711885

[60] Fogarty A, Brown S, Gartland D, Mensah F, Seymour M, Savopoulos P, et al. Psychosocial factors associated with adolescent depressive and anxiety symptoms during the COVID-19 pandemic. International Journal of Behavioral Development. 2022;46(4):308–19.

[61] Dean DJ, Tso IF, Giersch A, Lee HS, Baxter T, Griffith T, et al. Cross-cultural comparisons of psychosocial distress in the USA, South Korea, France, and Hong Kong during the initial phase of COVID-19. Psychiatry Res. 2021;295:113593. doi: 10.1016/j.psychres.2020.113593 33276269 PMC8087149

[62] Constantine MG, Chen EC, Ceesay P. Intake concerns of racial and ethnic minority students at a university counseling center: Implications for developmental programming and outreach. Journal of Multicultural Counseling and Development. 1997;25(3):210–8.

[63] Sun J, Rao N. Growing Up in Chinese Families and Societies. Int Perspect Early C. 2017;19:11–29.

[64] Shek DTL. SR. Parenting in Hong Kong: Traditional Chinese Cultural Roots and Contemporary Phenomena. Dordrecht: Springer; 2014.

[65] Pesce L, van Veen T, Carlier I, van Noorden MS, van der Wee NJA, van Hemert AM, et al. Gender differences in outpatients with anxiety disorders: the Leiden Routine Outcome Monitoring Study. Epidemiol Psych Sci. 2016;25(3):278–87. doi: 10.1017/S2045796015000414 25989916 PMC6998770

[66] Breslau J, Gilman SE, Stein BD, Ruder T, Gmelin T, Miller E. Sex differences in recent first-onset depression in an epidemiological sample of adolescents. Transl Psychiat. 2017;7. 10.1038/tp.2017.105 PMC5534940 28556831

[67] Hankin BL, Mermelstein R, Roesch L. Sex differences in adolescent depression: Stress exposure and reactivity models. Child Dev. 2007;78(1):279–95. doi: 10.1111/j.1467-8624.2007.00997.x 17328705

[68] Bogels SM, Brechman-Toussaint ML. Family issues in child anxiety: Attachment, family functioning, parental rearing and beliefs. Clin Psychol Rev. 2006;26(7):834–56. doi: 10.1016/j.cpr.2005.08.001 16473441

[69] Grano N, Karjalainen M, Ranta K, Lindgren M, Roine M, Therman S. Community-oriented family-based intervention superior to standard treatment in improving depression, hopelessness and functioning among adolescents with any psychosis-risk symptoms. Psychiat Res. 2016;237:9–16. doi: 10.1016/j.psychres.2016.01.037 26921045

[70] Simon Rice SH, Simon Blaikie, Katherine Monson, Rachel Stefaniak, Mark Phelan, Christopher Davey. Integrating family work into the treatment of young people with severe and complex depression: a developmentally focused model. Early intervention in psychiatry. 2016. doi: 10.1111/eip.12383 27696760

[71] Southwick SM, Vythilingam M, Charney DS. The psychobiology of depression and resilience to stress: Implications for prevention and treatment. Annu Rev Clin Psycho. 2005;1:255–91. doi: 10.1146/annurev.clinpsy.1.102803.143948 17716089

[72] Bryant RA, Harvey AG, Guthrie RM, Moulds ML. A prospective study of psychophysiological arousal, acute stress disorder, and posttraumatic stress disorder. J Abnorm Psychol. 2000;109(2):341–4. 10895573

[73] Davydov DM, Stewart R, Ritchie K, Chaudieu I. Resilience and mental health. Clin Psychol Rev. 2010;30(5):479–95. doi: 10.1016/j.cpr.2010.03.003 20395025

[74] Brown Ronald T LR. Family Functioning and Children’s Adjustment in the Presence of a Chronic Illness: Concordance Between Children with Sickle Cell Disease and Caretakers Families, Systems, & Health. 1999;17(2):165–79.

[75] Koelmel E, Hughes AJ, Alschuler KN, Ehde DM. Resilience Mediates the Longitudinal Relationships Between Social Support and Mental Health Outcomes in Multiple Sclerosis. Arch Phys Med Rehab. 2017;98(6):1139–48. doi: 10.1016/j.apmr.2016.09.127 27789238

[76] Steinhardt M, Dolbier C. Evaluation of a resilience intervention to enhance coping strategies and protective factors and decrease symptomatology. J Am Coll Health. 2008;56(4):445–53. doi: 10.3200/JACH.56.44.445-454 18316290

[77] Grant AM, Curtayne L, Burton G. Executive coaching enhances goal attainment, resilience and workplace well-being: a randomised controlled study. J Posit Psychol. 2009;4(5):396–407.

[78] Songprakun W, McCann TV. Effectiveness of a self-help manual on the promotion of resilience in individuals with depression in Thailand: a randomised controlled trial. Bmc Psychiatry. 2012;12. doi: 10.1186/1471-244X-12-12 22339984 PMC3298500

[79] Kumpfer KL, Magalhães C. Strengthening Families Program: An Evidence-Based Family Intervention for Parents of High-Risk Children and Adolescents. Journal of Child & Adolescent Substance Abuse. 2018;27(3):174–9.

[80] Kuhn ES, Laird RD. Family support programs and adolescent mental health: review of evidence. Adolescent Health, Medicine and Therapeutics. 2014;5:127–42. doi: 10.2147/AHMT.S48057 25177156 PMC4096456

[81] Wijsbroek SAM, Hale WW, Raaijmakers QAW, Meeus WHJ. The direction of effects between perceived parental behavioral control and psychological control and adolescents’ self-reported GAD and SAD symptoms. Eur Child Adoles Psy. 2011;20(7):361–71. doi: 10.1007/s00787-011-0183-3 21604192 PMC3135823

[82] Allison S, Roeger L, Martin G, Keeves J. Gender differences in the relationship between depression and suicidal ideation in young adolescents. Australian and New Zealand Journal of Psychiatry. 2001;35(4):498–503. doi: 10.1046/j.1440-1614.2001.00927.x 11531732

[83] Arslan G. Mediating role of the self–esteem and resilience in the association between social exclusion and life satisfaction among adolescents. Personality and Individual Differences. 2019;151:109514.

[84] Chervonsky E, Hunt C. Emotion regulation, mental health, and social wellbeing in a young adolescent sample: A concurrent and longitudinal investigation. Emotion. 2019;19:270–82. doi: 10.1037/emo0000432 29697988

